# IMiD-Free Interval and IMiDs Sequence: Which Strategy Is Better Suited for Lenalidomide-Refractory Myeloma?

**DOI:** 10.3390/life13112229

**Published:** 2023-11-20

**Authors:** Kazuhito Suzuki, Shingo Yano

**Affiliations:** Division of Clinical Oncology and Hematology, Department of Internal Medicine, The Jikei University School of Medicine, Tokyo 105-8461, Japan; yano@jikei.ac.jp

**Keywords:** multiple myeloma, immunomodulatory drug, proteasome inhibitor, anit-CD38 monoclonal antibody, lenalidomide-refractory

## Abstract

This review discusses immunomodulatory drug (IMiDs) sequencing and IMiD-free interval strategies for lenalidomide-refractory myeloma. IMiDs and proteasome inhibitors (PIs) improve clinical outcomes in patients with myeloma; however, refractoriness to lenalidomide, a category of IMiD, predicts poor outcomes. Next-generation IMiDs, such as pomalidomide, are effective even for lenalidomide-refractory myeloma. Therefore, an IMiD-sequencing strategy from lenalidomide to pomalidomide would be desirable. PIs are an antimyeloma therapeutic agent with another mode of action that might restore cereblon, a target of IMiDs; therefore, an IMiD-free interval via class switching from lenalidomide to PIs may be a promising alternative for lenalidomide-refractory myeloma. Additionally, the anti-CD38 monoclonal antibody is a key drug for salvage therapy in anti-CD38 monoclonal antibody-naïve patients. In clinical practice, safety profiles and social convenience can play important roles in the choice of combination therapy. In the future, the selection of optimal treatments should be based on the status of the immunological environment and genetic alterations. This review aims to discuss IMiDs sequencing and IMiD-free interval strategies for lenalidomide- refractory myeloma.

## 1. Introduction

Multiple myeloma is a hematopoietic tumor that is difficult to treat; however, the development of proteasome inhibitors (PIs), immunomodulators (IMiDs), and monoclonal antibody drugs (MoAbs) has improved clinical outcomes in myeloma patients. However, patients with myeloma who develop resistance to all three classes of agents show poor prognoses. Before MoAbs were available, several PI-plus-IMiD combination therapies were developed. However, these studies have revealed that PIs and IMiDs do not always act beneficially. Initial treatment with daratumumab (DARA), an anti-CD38 MoAb, in combination with PIs or IMiDs as initial therapy in transplant-naïve patients prolonged progression-free survival (PFS) and overall survival (OS) compared with those after conventional standard therapy in phase III ALCYONE and MAIA studies on transplant-ineligible newly diagnosed myeloma [1,2]. In addition, DARA-containing induction therapy was superior to DARA not-containing induction in transplant-eligible patients with newly diagnosed multiple myeloma (NDMM) who planned to receive PI not-included maintenance treatment [3,4], suggesting that long-term continuous PI and IMiD maintenance therapy might not be essential. Thus, after DARA is available for patients with NDMM, PI and IMiD combination therapy may be redundant, leading to the selection of PI or IMiD combined with DARA. However, lenalidomide (LEN) refractoriness remains an important factor affecting the therapeutic efficacy of both PI and IMiD (Table 1), even after another class of anti-myeloma therapeutics, such as anti-CD38 MoAbs, are available. According to several clinical trials, PI or next-generation IMID-containing therapies can improve clinical outcomes in patients with LEN-refractory myeloma patients [5,6,7,8,9,10,11,12,13]. In this review, we discuss treatment strategies for LEN-refractory myeloma that optimize the efficacy of IMiD-containing therapies by establishing a treatment period without IMiDs via administration of PIs, a so-called “IMiDs free interval”, or to continue IMiD-containing therapy, an “IMiDs sequence”.

## 2. Mechanism of Action in IMiDs

IMiDs bind to cereblon (CRBN) and exert various therapeutic effects; CRBN, together with damage-specific DNA binding protein 1 (DDB1), culllin-4A (Cul4A), and regulator of cullins 1 (Roc1), functions as an E3 ubiquitin ligase complex that converts recognized substrates upon binding to IMiDs. In myeloma cells, interferon regulatory factor 4 (IRF4), a transcription factor for myeloma cell growth, is suppressed via the degradation of Ikaros family zinc finger 1/3 (IKZF1/3), inducing a direct anti-myeloma effect [14]. In human myeloma cell lines in which *CRBN* gene expression was suppressed by shRNA, the antitumor effects of LEN and pomalidomide (POM) were reduced depending on their dose and the burden of *CRBN* gene reduction. In addition, IL-2 and TNF-α secretion was reduced in T cells co-cultured with a human myeloma cell line with CRBN knockout compared to those without CRBN knockout [15].

CRBN levels are decreased via proteasomal degradation after binding with IMiDs, whereas they are restored by the inhibition of proteasomal degradation by PIs [16]. Thus, therapeutic strategies for inhibiting proteasomal function with PIs and promoting proteasome-dependent IKZF1/3 degradation with IMIDs may be inconsistent. Thus, continuous administration of IMiDs decreases the CRBN level associated with their therapeutic effect while PIs may increase the CBRN level, suggesting that an “IMiDs free interval” with PI-containing therapy may improve the efficacy of subsequent IMiD-containing therapy via restoring CRBN.

## 3. Efficacy of Pomalidomide and Proteasome Inhibitors for Lenalidomide-Refractory Myeloma

POM, a second-generation IMiD, has been shown to be effective against LEN-refractory myeloma in basic research and clinical trials. Since CRBN plays an important role in the mechanism of action of IMiDs, *CRBN* gene mutations could induce resistance to both LEN and POM, but the frequency of *CRBN* gene mutations is low (10–30%) [17]. Additionally, E3 ubiquitin ligase and high karyopherin subunit alpha 2 (KNPA2) level mutation induced LEN-refractoriness as well [18,19], and might be potential for POM-refractoriness although there was no evidence; therefore, other mechanisms must underly the drug resistance of IMiDs. First, there was a difference in gene expression between myeloma cells treated with LEN and POM in a xenograft model [20]. Importantly, 1784 gene expressions changed by LEN were also changed by POM, whereas 94 genes were changed only by LEN and not by POM. In addition, only 64 common gene regulators were identified in myeloma cells resistant to both LEN and POM, although the numbers of downregulated genes in myeloma cells resistant to LEN and POM were 258 and 451, respectively. Thus, the genes associated with LEN resistance are different from those associated with POM. High *c-MYC* gene levels in myeloma cells are associated with LEN resistance [21]. In contrast, POM can suppress the *c-MYC* gene via proteasome-dependent degradation of AT-rich interaction domain 2 (ARID2), which is not a target molecule of LEN, suggesting that POM might be effective for a part of LEN-resistant myeloma cells [22]. Additionally, POM has therapeutic activity against TP53-related kinase via CRBN degradation, which is stronger than that of LEN [23]. POM can reduce the burden of plasmacytoma in severe combined immunodeficiency (SCID) mice with LEN-resistant myeloma cells, suggesting that POM is effective against LEN-resistant myeloma cells even when the immunological mechanism of action is excluded [20]. Moreover, regarding the immunological mechanism of action, counts of CD3^+^ and CD3^+^CD8^+^ T cells, which contribute an immune response for myeloma cells [24,25], were significantly increased with POM even after LEN resistance occurred while POM did not change CD4^+^ T cell level significantly [26]. In the APOLLO trial, low CD3^+^CD4^+^ T cell levels, especially CD3^+^CD4^+^ICOS (inducible T-cell co-stimulator)^+^ T cells as baseline, predicted a short PFS and low VGPR rate in the patients treated with DARA, POM and DEX [27]. On the other hand, LEN increased the CD4^+^ T cell and CD4^+^ /CD8^+^ levels in a phase 2 trial although CD8^+^ T cell and natural killer (NK) cell levels were not changed significantly [28]. Additionally, CD8^+^ central memory and effector memory T cell counts in the Rd group were significantly lower than those in the DARA-plus-Rd group using Cytometry by Time-Of Flight (CyTOF) in the POLLUX trial [29], and low CD8^+^ T cell count after three cycles of Rd predicted short PFS and poor therapeutic response from a Korean report [30], suggesting that low CD8^+^ T cell level might be associated with poor clinical outcome in the patients treated with LEN. Thus, POM has potential to improve immune microenvironment after Len-refractory myeloma patients, especially whose CD3^+^CD8^+^ T cell level was low. However, POM might not improve immunological activity in the LEN-refractory patients with low CD3^+^CD4^+^ T cell level.

PI has also been used against LEN-refractory myeloma in basic research. Recently, Maneix et al. reported that BOR reduced c-MYC protein levels via the stabilization of histone deacetylase 3 (HDAC3) and suppressed the proliferation of a c-MYC-overexpressing human myeloma cell line [31]. According to the TOURMALINE-MM1 trial, ixazomib, LEN, and dexamethasone improved PFS compared with Rd in patients with myeloma and a high expression of the *c-MYC* gene, although LEN-refractory myeloma patients were excluded from this trial [32]. Signal transducer and activator of transcription 3 (STAT3) activation via IL-6 autocrine is associated with LEN-resistance, and bromodomain for CRBN binding protein/E1A binding protein p300 (CBP/EP300) inhibition can release LEN-refractoriness via the STAT3-activated myeloma cell line [33]. BOR can suppress phosphorylation of STAT3 induced by IL-6 via myeloma cells and bone marrow stromal cell binding [34]. Thus, a class switch strategy targeting the *c-MYC* gene and STAT3 activation via IL-6 with PI-containing therapy may be a reasonable option for LEN-refractory myeloma. 

Finally, a common mechanism of refractoriness in PI and LEN was noted in the literature. For instance, mitogen-activated protein kinase (MEK)/extracellular signal-regulated kinase (ERK) pathway was shown to be related to common gene mutations concerning resistance to both LEN and POM, and overexpressed genes in the MEK/ERK pathway predicted resistance not only to IMIDs but also to BOR [17]. In fact, MEK inhibitors can reverse acquired resistance to IMIDs and PI [20]. Upregulated cyclin-dependent kinase (CDK) 6 induced resistance for not only BOR [35] but also LEN and POM [36], and CDK6 inhibitor could improve efficacy of BOR and LEN. Thus, CDK6 might be a key molecule to overcome BOR and LEN resistance, but palbociclib, a CDK4/6 inhibitor, could not show efficacy for myeloma patients in clinical trials [37]. Additionally, overexpressed runt-related transcription factors (RUNX)-1 and -3 [38,39,40], high-expressed heme oxygenase-1 (HO-1) [41,42], increasing sortilin 1 (SORT1)/lysosome-associated membrane protein 2 (LAMP2) [43], increasing extracellular vesicle [44,45,46,47] and overexpressed integrins β5 or 7 [48,49] also showed resistance for both LEN and PI. Thus, the class switch from IMiDs to PI cannot completely overcome LEN refractoriness if common pathological causes concerning resistance for both IMiDs and PI were present.

## 4. IMiDs Sequencing Strategy

We defined the “IMiDs sequence” as a sequential treatment strategy including IMiD-containing regimens. Prior to the development of POM-containing rescue chemotherapy, LEN played an important role in IMiD-based salvage chemotherapy. In addition, since LEN maintenance therapy was performed until disease progression as a standard care for transplant-eligible patients with NDMM, and Rd therapy was used until disease progression for transplantation-ineligible patients with NDMM in the FIRST trial, where salvage chemotherapy, including LEN, had to be one of the choices for second-line treatment, even if LEN was often used continuously [50,51]. However, LEN-resistant patients were excluded from the ASPIRE, TOURMALINE-MM1, ELOQUENT2, and POLLUX trials, which are pivotal clinical trials concerning three drug therapies with carfilzomib (CFZ), ixazomib, elotuzumab, and DARA in combination with Rd [52,53,54,55]. Therefore, a major issue regarding the continuous use of LEN in therapy has not been adequately validated. In a sub-analysis of second-line therapy in the FIRST trial, patients who received salvage therapy, including LEN, after continuous Rd therapy showed a low overall response rate and short time to next therapy (TTNT) compared with patients who received Rd therapy for 18 months [56]. Notably, the TTNT after continuous Rd was 15.5 months in patients treated with LEN-containing second-line therapy, which is not a much poorer outcome compared with the PFS (11.1 months) and time to progression (TTP) (13.4 months) in the MM-009 and -010 trials [57]. In addition, the PFS of LEN-containing salvage therapy combined with 56% of PI and 13% of DARA in patients who received LEN maintenance for 3 years or longer was significantly longer than that in patients who received LEN maintenance for 3 years or less [58], suggesting that LEN-containing second-line regimens might be effective even in patients with LEN refractoriness if initial LEN-containing therapy could continue for 3 years or longer. According to the IFM2009 and DETERMINATION trials, in autologous stem cell transplantation (ASCT) versus VRd (BOR, LEN plus DEX) followed by LEN maintenance therapy for 2 years or until disease progression, the OS between the ASCT and VRd arms in these trials [59,60] suggests that continuous LEN maintenance therapy might not be associated with poor clinical outcomes after recurrence compared with those in fixed-duration LEN maintenance therapy; however, there were no data on salvage therapies in these trials. 

According to several clinical trials, POM can be effective in LEN-refractory patients [11,12,13,22,27,61]. In the MM-003 trial, there were no significant differences in the overall response rate and PFS among the POM plus DEX (Pd) group with refractoriness to PIs, IMiDs, and the two classes of drugs [13]; however, the median PFS of 4.0 months in the Pd therapy group was unsatisfactory [13]. Pd provided insufficient PFS in the MM-003 trial because the median number of prior therapies was 5. Meanwhile, in the MM-014 cohort A, Pd therapy was associated with a median PFS of 9.6 months in patients with a median number of two prior regimens and LEN refractoriness just before Pd therapy [27], suggesting that early Pd therapy can play an important role in improving clinical outcomes. Additionally, in the ELOQUENT3 trial, which compared ELO plus Pd with Pd therapies, the OS of patients who became LEN-refractory during the last treatment was similar to that of the overall population [11]. In the POSEIDON trial, PFS and OS were similar in patients treated with Pd after LEN-containing and non-LEN-containing therapy as a last line [62]. Thus, Pd backbone regimens might not only improve but also overcome the negative impact of LEN-refractoriness even in the patients receiving LEN-containing therapy as a last line.

Anti-CD38 MoAb represents a new treatment option for LEN-refractory myeloma. Nijhof IS et al. demonstrated that DARA reduced the proliferation of LEN-refractory myeloma cells through ADCC [63]. In addition, DARA plus LEN significantly decreased the number of LEN-refractory myeloma cells compared to that with DARA monotherapy, and pretreatment of peripheral blood mononuclear cells (PBMC) [64] with LEN enhanced the anti-myeloma effect of DARA plus LEN. Isatuximab (ISA) showed not only ADCC but also a direct anti-myeloma effect, even in the presence of LEN or POM refractoriness [65]. In the SIRIUS [66] and TED10893 [67] trials, the therapeutic efficacies of the DARA and ISA monotherapies were independent of LEN refractoriness. Thus, the anti-CD38 MoAb may be effective for LEN-refractory myeloma. In the APOLLO trial, PFS in the DARA-plus-Pd (DPd) group was significantly longer than that in the Pd group. Notably, DPd therapy significantly prolonged PFS in patients who received 15 mg or more of LEN in the previous therapy, although there was no significant difference in PFS in patients who received 10 mg or less of LEN. In addition, DPd therapy showed a superior PFS response in patients who had received LEN for less than 1 year prior to therapy, although there was no significant difference with PFS in patients who received LEN for more than 1 year [12]. These two subgroup analyses revealed that the beneficial effects of the DARA add-on for Pd might be low in patients with LEN-sensitive relapsed myeloma, such that their LEN dose was low and LEN interval was long, because Pd might adequately improve clinical outcomes in this population. In MM-014 trial cohort B, a phase 2 trial investigating the efficacy of DPd therapy, the median PFS for all patients was 30 months, compared with 11.8 months in the APOLLO study [27]. However, PFS in LEN-refractory relapsed patients was 22 months, even when the median number of previous regimens was 1, and shorter than that in patients without refractoriness to LEN [13], suggesting that LEN refractoriness may also affect PFS in DPd therapy. In the ICARIA-MM trial, ISA plus Pd improved not only PFS but also OS compared to Pd in overall population, and the superiority of ISA plus Pd was observed even in the LEN-refractory RRMM patients [10,68]. Thus, anti-CD38 MoAb plus Pd can be a therapeutic option for LEN-refractory RRMM.

Recently, PI and IMiD combination treatment was shown to work synergistically via calpain-dependent IKZF1 cleavage [69] and stabilization of CRBN levels [70], suggesting that PI plus POM treatment might be a promising alternative for LEN-refractory myeloma. The OPTIMISMM trial revealed that POM plus Vd (PVd) was superior to Vd in patients with a treatment history of two or more cycles of LEN [5]. Basic research revealed that the combination of LEN and BOR acts via the stabilization of CRBN by BOR [70]. PFS in the PVd group was significantly longer than that in the Vd group for 71.2% of patients with refractoriness of LEN, and hazard ratios for the PFS of PVd compared to Vd were similar in all patients (HR 0.61) and limited LEN-refractory patients (HR 0.65), although the actual PFS in all patients treated with PVd was slightly short compared to that in the LEN-refractory patients (11.2 and 9.53 months, respectively). Thus, PVd is a good option for patients with LEN-refractory myeloma, but even PVd is not adequately effective against LEN refractoriness. Furthermore, CFZ, POM, and DEX might be an option for LEN-refractory myeloma, although phase III clinical trials are still needed [71,72]. Finally, according to the US registry data analysis, PFS in patients treated with POM-containing therapy was longer than that in patients treated without POM as second-line therapy, including LEN, although three drug therapies, DPd and CFZ plus Pd, were included in the POM-containing therapy group [73].

In summary, the “IMiDs sequence” may also be an adequate treatment option in modern clinical settings, although LEN refractoriness can be often associated with poor clinical outcomes in patients receiving LEN- and POM-containing salvage therapies, and many POM-containing therapies have been developed as salvage therapy. Notably, a POM-containing three-drug therapy was selected as part of the control arm in recent phase 3 clinical trials for LEN-refractory RRMM patients [74].

## 5. IMiDs Free Interval

A single-center retrospective study from Greece reported that an IMiD-free interval of 18 months was an important predictor of survival benefits with Pd therapy [75]. The immediately preceding LEN dose did not affect the therapeutic effect of Pd therapy, suggesting that LEN resistance can be determined regardless of the LEN dose and may reduce POM sensitivity. This study noted the importance of a treatment strategy that discontinues IMiD-based therapy to establish an “IMiDs free interval”. The optimal cutoff for the IMiD-free interval remains unclear because no large-scale prospective clinical trial has yet addressed this issue. Additionally, few clinical trials of salvage therapy achieved a median PFS of 18 months or longer in patients receiving non-LEN-containing regimens.

PIs can restore CRBN by blocking its proteasome-dependent degradation [16]. However, whether the optimal IMiD-free interval is 18 months or longer when using only PI remains controversial. The safety of 56 mg/m^2^ CFZ twice weekly was reported in a phase I clinical trial, which inspired the development of high-dose CFZ-containing therapies [76]. The high-dose CFZ therapy can target both the conventional β5 subunit as well as the β2 subunit, which causes BOR resistance, which represents a novel mechanism of action for PI therapy that has not been recognized in BOR, ixazomib, and low-dose CFZ [77]. The superior efficacy of high-dose CFZ plus DEX (Kd) therapy over Vd therapy was reported in the ENDEAVOR trial, with a median PFS of 18.7 months in all patients [6], suggesting that salvage chemotherapy without IMiDs can also achieve a PFS of 18 months or longer. However, subgroup analysis in the Kd group confirmed a trend toward shorter PFS in LEN-refractory patients, suggesting that LEN refractoriness is associated with PI sensitivity and remains an important issue in the treatment of relapsed/refractory multiple myeloma (RRMM). 

Three drug therapies combining anti-CD38 MoAbs, such as DARA and ISA, and Kd therapy significantly prolonged the median PFS to 28.5 and 35.7 months in all patient groups, respectively, compared to that with Kd therapy in the CANDOR and IKEMA trials [8,9]. Notably, the PFS in the DARA-plus-Kd group was similar between LEN-refractory and non-refractory patients (28.1 and 28.6 months) in the CANDOR trial [78], and the PFS of the LEN-refractory patients treated with ISA plus Kd was significantly longer than for those treated with Kd monotherapy in the long follow-up analysis of the IKEMA trial as well [79]. Finally, the MRD negativity rate in LEN-refractory patients was similar to that in all patients after treatment with ISA plus Kd (24.6% and 29.6%) [80]. We believe that these three-drug combination therapies can provide a long-term IMiD-free interval compared with high-dose Kd monotherapy, especially in LEN-refractory patients.

## 6. “IMiDs Free Interval” vs. “IMiDs Sequence”

It remains unclear whether the “IMiDs free interval” or “IMiDs sequence” is a superior therapy. However, if the disease becomes refractory to LEN-containing initial therapies, it is likely to remain susceptible to PI, anti-CD38 MoAb, or both as second-line therapy [81]. For this line of treatment, it is important to determine whether the patient has true double refractory multiple myeloma (DRMM). For example, in the VRd arm of the SWOG s0777 trial [82], if disease progression occurred within 6 months of treatment initiation, it was considered to be DRMM, and a POM-based regimen was selected. In contrast, disease progression after more than 6 months of LEN-containing therapy may reflect an IMiD-sensitive myeloma. According to a Mayo Clinic report, the PFS from second-line treatment (2nd PFS) was similar among PI, IMiDs, and PI plus IMiDs with salvage therapy after ASCT followed by lenalidomide maintenance [83]. In the patients treated with the IMiD-containing regimen, the second PFS in the patients treated with pomalidomide was significantly longer than those with lenalidomide. A subgroup analysis of the APOLLO trial revealed that low-dose and long-duration LEN administration, such as LEN maintenance therapy, resulted in a relatively high therapeutic efficacy of Pd. Additionally, a retrospective analysis showed that 3 years or longer of LEN maintenance therapy did not suppress the therapeutic efficacy of LEN-containing salvage therapy as described above. These findings suggest LEN-refractory myeloma should be divided into “myeloma cell with resistance for LEN” and “worsening bone marrow microenvironment during LEN” to select optimal salvage therapies after LEN refractoriness is established. However, methods to identify the cause of LEN refractoriness in clinical practice are ineffective. The immune microenvironment may influence the immunomodulatory effects of POM as well as anti-CD38 MoAb. Therefore, “IMiDs sequence” is worth considering not only for myeloma cells but also for the immune microenvironment and the combined effect with MoAbs.

Cytogenetic risk was associated with clinical outcomes in both newly diagnosed MM and RRMM [84,85]. The frequency of 1q21 abnormality and del17p were increased in RRMM compared to those at diagnosis, and these cytogenetic abnormalities worsened survival time in RRMM [85]. Pomalidomide has potential efficacy to improve clinical outcomes for RRMM with del17p according to the results in MM-003 and ICARIA trials [86,87]. According to the findings of the IKEMA, FORTE, and ENDURANCE trials, proteasome inhibitors, particularly CFZ, helped patients with 1q21 abnormalities [88,89,90,91]. Thus, cytogenetic abnormality might help select salvage therapy for RRMM with lenalidomide refractoriness.

Anti-CD38 MoAb-containing salvage therapy should be the standard of care for patients with anti-CD38 MoAb-naive RRMM. Regarding exposure and safety, PI might be a more suitable option than POM in combination with the anti-CD38 MoAb because the incidence of dose reduction due to hematological toxicity is more frequent with anti-CD38 MoAb plus POM than that with PI [12,52]. In addition, CD38 downregulation may induce increased coronary flow and prevent cardiac toxicity caused by PI [92,93]. In terms of therapeutic activity, the PI and anti-CD38 MoAb combination can enhance ADCC by downregulating HLA class A [94,95] and complement-dependent cytotoxicity (CDC) by downregulating CD55 and CD59 [96]. Finally, the PI plus anti-CD38 MoAb combination improved CAM-DR owing to CD31 and CD38 interactions [97]. Thus, the anti-CD38 MoAb plus PI combination could be administered without dose reduction due to hematological toxicity compared with that in the POM combination, and also induce therapeutic effects.

After exposure to both LEN and anti-CD38 MoAb, PI-containing therapy might be the mainstream salvage therapy. The combination of PI and Pd can be a good option, when possible. However, a continuous anti-CD38 MoAb strategy has not yet been established in clinical trials [98,99]. Finally, we must select a suitable salvage therapy in PI- or POM-based regimens that considers complications such as cytopenia, cardiac complications, hypertension, chromosomal abnormalities, and the frequency of hospital visits. The advantages and disadvantages of IMiD sequencing and IMiD-free intervals are shown in Table 2.

In our clinical practice, “IMiDs free interval” strategy, mainly Kd containing therapy, is generally the first choice as salvage therapy for last line LEN refractory RRMM when cardiovascular disfunction and peripheral neuropathy are not severe, and the patient can visit our clinic frequently from social and economic points of view. Meanwhile, if the patients have some difficulty with Kd-based therapy, the “IMiDs sequence” strategy is also an alternative option. Anti-CD38 MoAb plus Pd is the first choice if anti-CD38 MoAb is naïve. We have to pay a tension for cytopenia and infection during anti-CD38 MoAb plus Pd, especially within the first several months. For the patients with exposure of anti-CD38 MoAb, ELO plus Pd can be an option even if cytopenia is severe because the incidence of cytopenia in ELO plus Pd was lower than those in Pd according to ELOQUENT3 trial.

## 7. Future Directions

**Figure 1:**
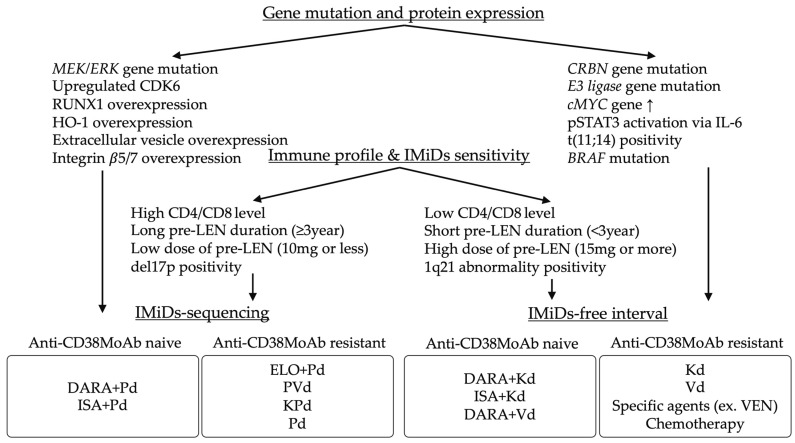
Stratifications between IMiDs-sequencing and IMiDs-free interval based- treatment considering gene mutation, protein expression, immune profile, and IMiDs sensitivity. MEK/ERK, mitogen-activated protein kinase/extracellular signal-regulated kinase; CDK6, cyclin dependent kinase 6; RUNX1, runt-related transcription factors-1; HO-1, heme oxygenase-1; CRBN, cereblon; STAT3, signal transducer and activator of transcription 3; IL-6, interleukin-6; BRAF, v-raf murine sarcoma viral oncogene homolog B1; LEN, lenalidomide; IMiDs, immunomodulatory drugs; MoAb, monoclonal antibody; DARA + Pd, daratumumab, pomalidomide plus dexamethasone; ISA + Pd, isatuximab, pomalidomide plus dexamethasone; ELO + Pd, elotuzumab, pomalidomide plus dexamethasone; PVd, pomalidomide, bortezomib, plus dexamethasone; KPd, carfilzomib, pomalidomide plus dexamethasone; Pd, pomalidomide plus dexamethasone; DARA + Kd, daratumumab, carfilzomib plus dexamethasone; ISA + Kd, isatuxmab, carfilzomib plus dexamethasone; DARA + Vd, daratumumab, bortezomib plus dexamethasone; Kd, carfilzomib plus dexamethasone; Vd, bortezomib, plus dexamethasone; VEN, venetoclax.

The informed application of IMiD sequencing and IMiD-free intervals requires further advances in gene mutation analysis using next-generation sequencing and investigation of the immune microenvironment using mass spectrometry. For instance, if the cause of LEN refractoriness worsens immunological issues, especially reducing CD8^+^ T cell count, POM might be a suitable option for improving the immunological environment. Additionally, low CD4^+^ T cell level predicted poor outcomes in the patients treated with POM, suggesting that high CD4/CD8 level might be a prognostic factor for the immunological efficacy of POM. However, if the cause of LEN refractoriness is a gene mutation, such as CRBN, PI might be a suitable option. Currently, the detection of new drugs for suitable targets, such as B-cell mature antigen (BCMA) [103,104,105], G protein-coupled receptor class C group 5D (GPRC5D) [106,107], and Fc receptor-homolog 5 (FcRH5) [108], is developing. Additionally, specific gene mutations such as v-raf murine sarcoma viral oncogene homolog B1 (BRAF) and cytogenetic abnormalities such as t(11;14) are identified in myeloma cells, where BRAF inhibitors and venetoclax may be effective [109,110,111,112,113]. Therefore, suitability of future therapies can be improved through the characterization of gene mutations, the IMiD sensitivity considering the previous LEN duration and dose, the immunological environment, and the presence of new targeting molecules. Finally, social issues, such as frequency of clinical visits, distance from home to clinic, and care givers’ concerns, should be key in selecting a therapeutic regime. Treatment options for LEN-refractory MM, considering genetic mutation and protein expression, immune profile, and IMiDs sensitivity, are shown in Figure 1. 

## 8. Conclusions

Many salvage chemotherapies, including POM, PIs, and anti-CD38 MoAb, are now available as early-line treatments. Both the “IMiDs free interval” and “IMiDs sequence” are innovative concepts in the treatment of potentially LEN-refractory patients with MM. The “IMiDs free interval” makes sense when considering CRBN expression and has been successfully applied in various guidelines, but further evidence is required for its clinical application. The “IMiDs sequence” can be a therapeutic option even in LEN-refractory patients, but it might not be optimal considering the CRBN concerns. If the “IMiDs sequence” strategy is selected, POM should be administered with consideration for enhancing immunological activity and clinical evidence in the RRMM with LEN-refractoriness. In the future, the characteristics of myeloma cells and state of the immune microenvironment should be considered when selecting the most appropriate therapy for each patient.

## Figures and Tables

**Table 1:** Summary of clinical trials for the proteasome inhibitor or pomalidomide containing regimens in the lenalidomide-refractory myeloma patients.

		PFS (Experimental)		PFS (Control)		HR	
		All	LEN-ref	All	LEN-ref	All	LEN-ref
OPTIMISMM [5]	PVd vs. Vd	11.2	9.53	7.10	5.59	0.61	0.65
ENDEAVOR [6]	Kd vs. Vd	18.7	8.6	9.4	6.6	0.53	0.86
CASTOR [7]	DVd vs. Vd *	16.7	9.3	7.1	4.4	0.31	0.36
CANDOR [8]	DKd vs. Kd	28.6	28.1	15.2	11.1	0.63	0.49
IKEMA [9]	ISA + Kd vs. Kd	NE	NE	19.5	15.7	0.53	0.598
ICARIA [10]	ISA + Pd vs. Pd	11.5	11.4	6.47	5.59	0.596	0.59
ELOQUENT3 [11]	EPd vs. Pd	10.3	NA	4.7	NA	0.54	0.56
APOLLO [12]	DPd vs. Pd	12.09	9.89	7.03	6.54	0.63	0.64
MM-014 [13]	DPd	30.8	21.8	NA	NA	NA	NA

* Lenalidomide-refractory as last line treatment. PFS, progression free survival; HR, hazard ratio; LEN-ref, lenalidomide-refractory; PVd, pomalidomide, bortezomib plus dexamthasone; Vd, bortezomib plus dexamthasone; Kd, carfilzomib plus dexamthasone; DVd, daratumumab, bortezomib plus dexamthasone; DKd, daratumumab, carfilzomib plus dexamthasone; IsaKd, isatuximab, carfilzomib plus dexamthasone; IsaPd, isatuximab, pomalidomide plus dexamthasone; Pd, pomalidomide plus dexamthasone; EPd, elotuzumab, pomalidomide plus dexamthasone; DPd, daratumumab, pomalidomide plus dexamthasone.

**Table 2:** PROS and CONS for IMiDs-sequencing and IMiDs-free interval.

	IMiDs-Sequencing	IMiDs-Free Interval
PROS	There was several evidences about clinical efficacy of POM after LEN refractory, such as MM-003, MM-014, APPOLO, ICARIA-MM, ELOQUENT3, OPTIMISMM, and KPd trials [5,10,11,12,13,71,72,73,86]. POM can improve immunological microenvironment even after LEN refractory [24,25,26]. POM might be effective for myeloma cells with high level cMYC gene, which is related with LEN refractory [22]. High IMiDs sensitivity might predict good clinical outcome in POM [12,58]. Anti-CD38 MoAb plus POM can improve clinical outcome even for LEN refractory RRMM in APOLLO, MM-014, and ICARIA-MM trials [10,12,13,27].	Class switch strategy is recommended according to several clinical guidelines [100,101,102]. PI can restore CRBN, inducing that clinical outcome can get better using IMiDs in next treatment [16]. PI might be effective for myeloma cells with high level cMYC gene and activated STAT3, which is related with LEN refractory [31,32,33,34]. Anti-CD38 MoAb plus PI can improve clinical outcome even for LEN refractory RRMM in CANDOR, IKEMA, and CASTOR trials [7,8,9].
CONS	CRBN and E3 ubiquitin ligase gene mutation was associated with refractoriness for both LEN and POM [17,18,19,20]. IMiDs-free interval might improve clinical outcome of POM containing treatment [75].	There was a few evidences about class switch strategy and IMiDs-free interval. Therapeutic efficacy of PI decreases after LEN refractory because PI and LEN can share same mechanism for refractoriness, such as MEK pathway [20,35,36,37,38,39,40,41,42,43,44,45,46,47,48,49].

POM, pomalidomide; LEN, lenalidomide; CBRN, celebron; PI, proteasome inhibitor; MoAb, monoclonal antibody.

## Data Availability

Not applicable.
